# A Method for Manufacturing Oncological Phantoms for the Quantification of 18F-FDG PET and DW-MRI Studies

**DOI:** 10.1155/2017/3461684

**Published:** 2017-09-07

**Authors:** Francesca Gallivanone, Irene Carne, Matteo Interlenghi, Daniela D'Ambrosio, Maurizia Baldi, Daniele Fantinato, Isabella Castiglioni

**Affiliations:** ^1^Institute of Molecular Bioimaging and Physiology, National Research Council (IBFM-CNR), Milan, Italy; ^2^Medical Physics Unit, IRCCS Fondazione S. Maugeri, Pavia, Italy; ^3^Department of Diagnostic Imaging, IRCCS Fondazione S. Maugeri, Pavia, Italy

## Abstract

The aim of this work was to develop a method to manufacture oncological phantoms for quantitation purposes in 18F-FDG PET and DW-MRI studies. Radioactive and diffusion materials were prepared using a mixture of agarose and sucrose radioactive gels. T2 relaxation and diffusion properties of gels at different sucrose concentrations were evaluated. Realistic oncological lesions were created using 3D-printed plastic molds filled with the gel mixture. Once solidified, gels were extracted from molds and immersed in a low-radioactivity gel simulating normal background tissue. A breast cancer phantom was manufactured using the proposed method as an exploratory feasibility study, including several realistic oncological configurations in terms of both radioactivity and diffusion. The phantom was acquired in PET with 18F-FDG, immediately after solidification, and in DW-MRI the following day. Functional volumes characterizing the simulated BC lesions were segmented from PET and DW-MRI images. Measured radioactive uptake and ADC values were compared with gold standards. Phantom preparation was straightforward, and the time schedule was compatible with both PET and MRI measurements. Lesions appeared on 18F-FDG PET and DW-MRI images as expected, without visible artifacts. Lesion functional parameters revealed the phantom's potential for validating quantification methods, in particular for new generation hybrid PET-MRI systems.

## 1. Introduction

*In vivo* molecular imaging techniques, such as Computed Tomography (CT), Positron Emission Tomography (PET), and Magnetic Resonance Imaging (MRI)—in particular Diffusion Weighted MRI (DW-MRI)—are commonly used for the diagnosis, prognosis, and therapy monitoring of many diseases. These techniques are inherently quantitative; thus, advanced image processing methods for extracting quantitative parameters have been developed for use as surrogate disease biomarkers. In addition, in recent years, advanced image processing methods, such as texture and shape analyses, able to extract intratumor phenotypic heterogeneity at image level, have emerged as diagnostic and predictive biomarkers for the characterization of oncological lesions and for prediction of single patient prognosis (e.g., [1]). Quantification strategies to extract biomarkers from* in vivo* molecular imaging techniques need to be validated in order to evaluate their performance, accuracy, and robustness, while comparing the values obtained by the application of image processing with actual values (gold standard, GS).

Even if, at a first level, the evaluation of quantitative strategies for volume and shape descriptors could be performed on patient data by comparison with qualitative features and manual contouring defined by a physician, this validation strategy is suboptimal. In fact, both qualitative evaluations of shape descriptors and manual contouring are operator-dependent, not ensuring an accurate GS for validation.

Simulations by Monte Carlo methods have been extensively applied to clinical molecular imaging in order to deal with a variety of problems that are difficult to study by an experimental or analytical approach [2, 3]. Digital anthropomorphic phantoms, even derived from real patient studies, can be defined using Monte Carlo methods, with the advantage of controlling the ground truth and the clinical situation to be represented. However, even if these methods achieved noteworthy results [4, 5], the simulation of all the noise and signal components occurring in clinical images, especially for whole-body studies, still represents a limitation [6].

Other approaches for the validation of image processing methods include the use of synthetic image datasets obtained by experimental measurements in phantoms, simulating realistic conditions, for example, including oncological lesions characterized by a functional signal with irregular spatial distribution and heterogeneous signals. In experimental synthetic datasets, GS data, such as lesion volume and functional content, are known, thus allowing us to test the accuracy and robustness of image processing methods. Oncological phantoms consist of outer plastic shells in appropriate anatomical shapes containing objects of a known volume and shape that mime oncological lesions. These can be filled with materials simulating the properties of oncological lesions as depicted by the particular imaging technology (e.g., radiopaque materials [7], radioactive solutions [8], and tissue-equivalent diffusivity materials [9]). The possibility of accurately reproducing nonspherical and heterogeneous lesions is challenging, since these are common characteristics in real oncological clinical studies which often jeopardize the feasibility and accuracy of image quantification methods. 3D printing is a low-cost tool to produce custom-made phantoms from clinical settings and can create a lesion with complex shape [10]. However, in order to create biological unevenness or necrosis, causing nonuniform signal responses on images, appropriate strategies using specific materials are needed.

The aim of this work was thus to develop a method to manufacture oncological phantoms, including nonspherical and nonuniform objects, which mime realistic oncological lesions. These phantoms were developed for validation of image quantification strategies in both ^18^F-FDG PET and DW-MRI studies and are not intended to be used for quality control testing the characteristics and accuracy of scanners.

## 2. Materials and Methods

### 2.1. Oncological Phantom

Phantom material is based on a mixture of agarose and sucrose gels. Aqueous sucrose solutions have been proposed to measure and control the Apparent Diffusion Coefficient (ADC) in DW-MRI [11, 12]. However, to our knowledge, no phantoms have been developed that are compatible with both PET and DW-MRI.

Agarose (e.g., UltraPure™ Agarose, Invitrogen Life Technologies, UK), sucrose (Sigma-Aldrich, UK), and sodium chloride (Sigma-Aldrich, UK) are mixed in a beaker with deionized water and stirred at room temperature. A fixed quantity of 9 g/L of sodium chloride is used, while agarose is maintained at a 2% w/v concentration, and sucrose can vary depending on the desired ADC values (the higher the sucrose concentration, the higher the ADC values).

The beaker with the mixture is placed in a microwave for agarose complete dissolution (12 min/300 mL at 450 W). The hot mixture is then placed on a shaker (e.g., Grant, Mini Rocker PMRI-30, UK) and the drop in temperature is monitored.

To create oncological lesions for both DW-MRI and ^18^F-FDG PET studies, ^18^F-FDG at the desired radioactivity concentration is added to the gel mixture. Radioactivity concentration of the gel mixture can be measured by a gamma counter to provide the GS.

**Figure 1:**
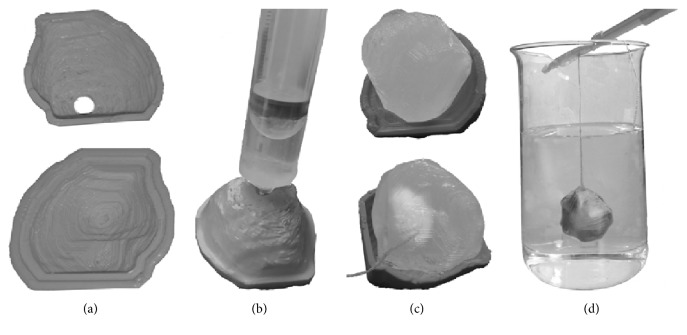
(a) 3D-printed shell; (b) shell filling procedure; (c) solidified extracted gel, with and without necrosis; (d) suspended lesion in solidifying phantom background.

Molds simulating realistic irregular oncological lesions are manufactured in plastic using a 3D printer. Molds of different shapes and volumes are derived by segmenting volumes of real oncological lesions from MRI and ^18^F-FDG PET images [10], printed as two empty shells, closed, and filled through a small hole with the radioactive gel using a syringe (Figures 1(a) and 1(b)).

Once solidified, gel is extracted from the mold (Figure 1(c)). Gel net weight can be measured using an analytical balance, and gel volume can be obtained using the characterized gel density to provide GS. This avoids errors in volume estimation due to errors in shell filling. The solidified gel is sealed with a thin plastic Parafilm to prevent any release of ^18^F-FDG or sucrose.

Oncological lesions with necrotic portions are created suspending portions of ~1 cc of solidified nonradioactive gel mixture (e.g., with 2% agarose and 1% sucrose), sealing the molds with Parafilm before filling.

The phantom background is prepared using a gel mixture at the desired radioactivity concentration (lower than the lesion radioactivity concentration for realistic oncological studies). The background mixture is poured into a beaker. When the temperature of the background mixture drops below 50°C, simulated lesions are suspended into the background mixture (Figure 1(d)). The phantom is left at room temperature inside a fume hood.

After preparation, the phantom can be immediately used for PET studies, and for DW-MRI the following day, after radioisotope decay.

Figure 1 shows the experimental setup for the phantom preparation. The same mold was filled with a uniform radioactive gel (Figure 1(c)) and nonuniform radioactive gel (to simulate a lesion with necrosis).

### 2.2. Exploratory Feasibility Study

PET measurements were performed on a Discovery-STE PET/CT system, according to the clinical oncological protocol, including a low-dose CT scan (40 mA) for patient positioning, a CT scan (140 keV, 150 mA, 10 sec), and 3D PET scans (2.5 min/scan for adjacent bed positions) for the radiation attenuation correction and radioactivity distribution acquisition, respectively. PET images were reconstructed by a 3D ordered subset expectation maximization algorithm (OSEM, 28 subsets, 2 iterations, 5.14 mm Gaussian postsmoothing) with corrections for random, scatter, and attenuation incorporated into the iterative process.

MRI measurements were performed with a 3T scanner (Discovery MR750, GE Healthcare) equipped with a dedicated double breast 8-channel coil. The MRI clinical protocol included T2-weighted sequences and DW. The DW sequences used a spin echo single-shot echo-planar sequence (SE-EPI) on the axial plane (TR/TE = 4800/61 ms, matrix = 192 × 192, FOV = 480 × 288 mm, number of slices = 40, slice thickness = 4.5 mm, NEX = 16, acquisition time = 5 : 12 min) and diffusion gradients applied along the *x*-, *y*-, and *z*-axes (DIFF = ALL) with *b* values of 0–500, 0–750, and 0–1000 s/mm^2^.

Both PET and MRI images were processed with algorithms developed in Matlab 2015b.

#### 2.2.1. MRI Characterization of the Gel Mixture

Temporal stability of T2 relaxation time and diffusion characteristics of the gel mixture were assessed. Specifically, 300 mL of gel mixture was poured into a 500 mL plastic beaker with 2% agarose and 12 different sucrose concentrations (1%, 5%, 10%, 15%, 20%, 25%, 30%, 32.5%, 35%, 37.5%, 40%, and 42.5%).

When the temperature of each gel mixture dropped below 80°C, 6 g/L of diazolidinyl urea (Sigma-Aldrich, UK) was added. When the temperature dropped to 50°C, each gel mixture was then removed from the shaker and left to solidify at room temperature. The gel was extracted from the plastic beaker and an MR scan was acquired.

T2 maps were generated, starting from the T2 values, using monoexponential, nonlinear fittings to the appropriate equations describing the decay of the T2 signal. ADC maps were generated with two diffusion *b* values as in the clinical settings described above, and with other *b* values (0, 500, 750, and 1000 s/mm^2^). Monoexponential nonlinear fittings to the appropriate equations were used, describing the signal in DWI acquisitions. Four different sets of ADC maps were obtained.

A Volume-of-Interest (VOI) of fixed dimension (63 cc) was placed on both T2 and ADC maps of each gel mixture, for each sucrose concentration, thus excluding possible artifacts at the interface between air and gel mixture. Average T2 and ADC values within VOI were calculated and evaluated as a function of sucrose concentration (using a nonparametric fitting). Temporal stability of T2 relaxation and diffusion properties (ADC) were evaluated on the basis of their variation within a short-term period of 5 weeks, calculating coefficients of variation (CVs).

#### 2.2.2. Proof-of-Concept Study

We tested the oncological phantom in a breast cancer (BC) configuration. Lesion volumes ranged from 2.3 to 11.5 cc. Sucrose concentrations from 25% to 42.5% were used to simulate diffusion properties of high cellularity BC tissues. ^18^F-FDG radioactive concentrations from 0.02 to 0.47 MBq/cc were used according to those measured in real BC lesions from ^18^F-FDG PET images. Phantom background was prepared using 500 cc gel mixture at radioactivity concentrations ranging from 0.02 to 0.002 MBq/cc, covering lesion-to-background ratios from 3 to 30, according to those measured in real BC ^18^F-FDG PET studies.

The simulated BC lesions were segmented on both PET images and ADC maps, using validated semiautomatic segmentation methods. Metabolic Tumor Volume (MTV) was obtained from PET using a fully automatic segmentation method based on adaptive thresholding [10]. ADC volume (*V*_ADC_) was obtained from DW-MRI using a fixed threshold-based segmentation method [13].

Measured radioactivity concentration and ADC were compared with their GS values.

## 3. Results

### 3.1. Oncological Phantom

Our procedure for phantom preparation was straightforward, and the time schedule was compatible in particular with PET measurements, since the time required for gel solidification varied from a few minutes to half an hour depending on gel quantity.

### 3.2. Exploratory Feasibility Studies

#### 3.2.1. MRI Characterization of the Gel Mixture

**Figure 2:**
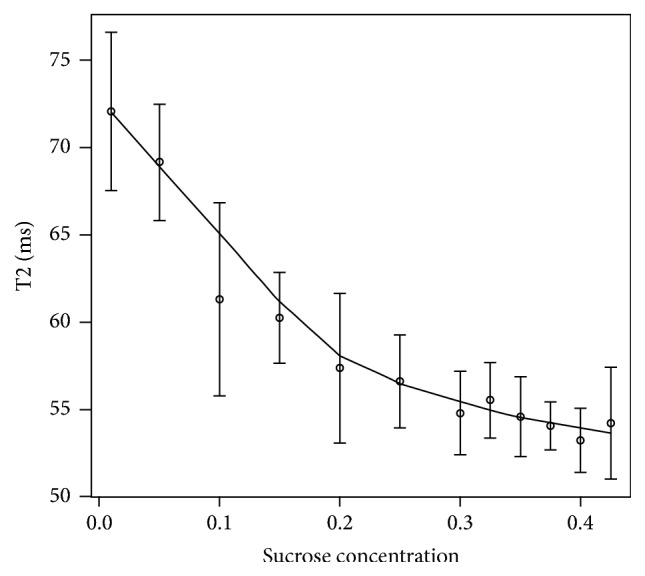
T2 calibration curve averaged on 5 time measurements.

Considering the MRI characterization of the gel mixture, Figure 2 shows T2 values as a function of sucrose concentration. Mean T2 and standard deviation over 5 different temporal measurements (performed at 5 consecutive weeks) are shown.

Mean T2 values ranged from 50.7 ms to 80.4 ms, compatible with T2 relaxation times of breast tissues. This range is compatible with that obtained by Lavdas et al. (2013) [11]. A dependence on sucrose concentration was found, as expected (nonparametric fitting *R*-square = 0.98).

T2 CVs (over 5 different temporal measurements) ranged from 2.5% (1% w/v sucrose concentration) up to 9.0% (42.5% w/v sucrose concentration).

**Figure 3:**
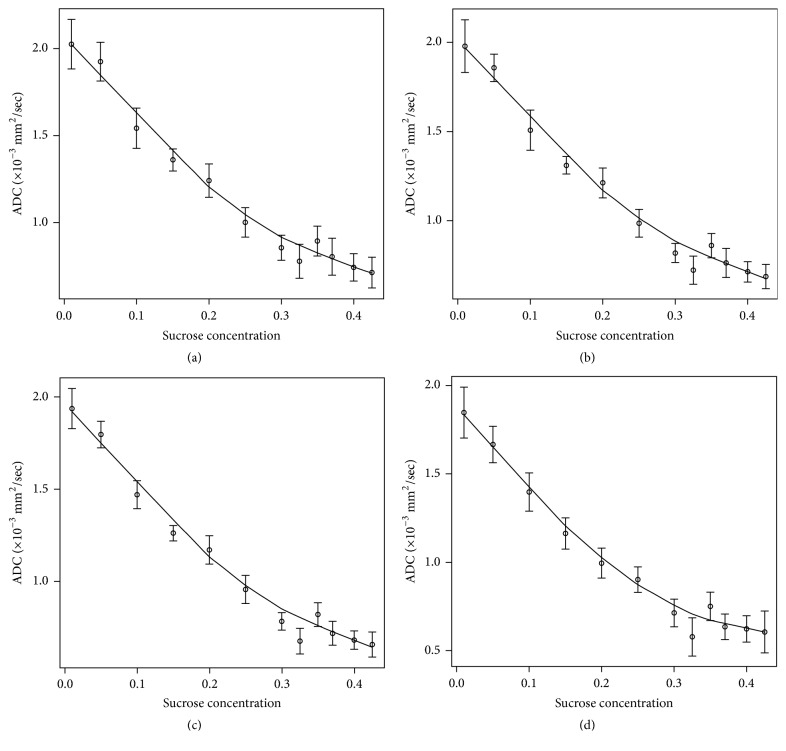
ADC as a function of sucrose concentration using (a) *b* = 0 s/mm^2^ and *b* = 500 s/mm^2^, (b) *b* = 0 s/mm^2^ and *b* = 750 s/mm^2^, (c) *b* = 0 s/mm^2^ and *b* = 1000 s/mm^2^, and (d) all the four *b* values (*b* = 0, 500, 750, and 1000 s/mm^2^).

Figure 3 shows ADC values as a function of sucrose concentration (mean ADC within VOI). Results are shown for the first measurement out of five (the one performed the first week): for *b* = 0 s/mm^2^ and *b* = 500 s/mm^2^ (Figure 3(a)), for *b* = 0 s/mm^2^ and *b* = 750 s/mm^2^ (Figure 3(b)), for *b* = 0 s/mm^2^ and *b* = 1000 s/mm^2^ (Figure 3(c)), and for all *b* values, *b* = 0, 500, 750, and 1000 s/mm^2^ (Figure 3(d)).

Mean ADC values ranged from 0.5*∗*10^−3^ mm^2^/sec to 2*∗*10^−3^ mm^2^/sec, depending on the method used to obtain ADC maps. A dependence on sucrose concentration was found, as expected (nonparametric fitting *R*-square from 0.92 to 0.98).

ADC CVs (over 5 different temporal measurements) ranged from 2.5% (1% sucrose concentration) to 22.8% (42.5% sucrose concentration), with an even worse temporal instability of ADC than T2 at a high sucrose concentration. This trend is compatible with the water evaporation observed across weeks in the gel mixture (observed by measuring its weight) and impacts on the sucrose concentration over time (variation in sucrose concentration up to 10%).

Despite T2 CV being less than 10%, the temporal instability of ADC at a high sucrose concentration suggests using lesions with a high sucrose concentration within three weeks of their preparation (in this case CV is up to 10% as for T2).

Calibration curves show that, using sucrose concentration smaller than 20% w/v, simulated ADC values are similar to those found in normal breast tissue (ADC > 10^−3^ mm^2^/sec), while sucrose concentration greater than 20% w/v could be used to simulate ADC values of malignant oncological lesions.

#### 3.2.2. Proof-of-Concept Study

The feasibility of the phantom was proven in a wide range of BC diffusion and radioactivity configurations.

**Figure 4:**
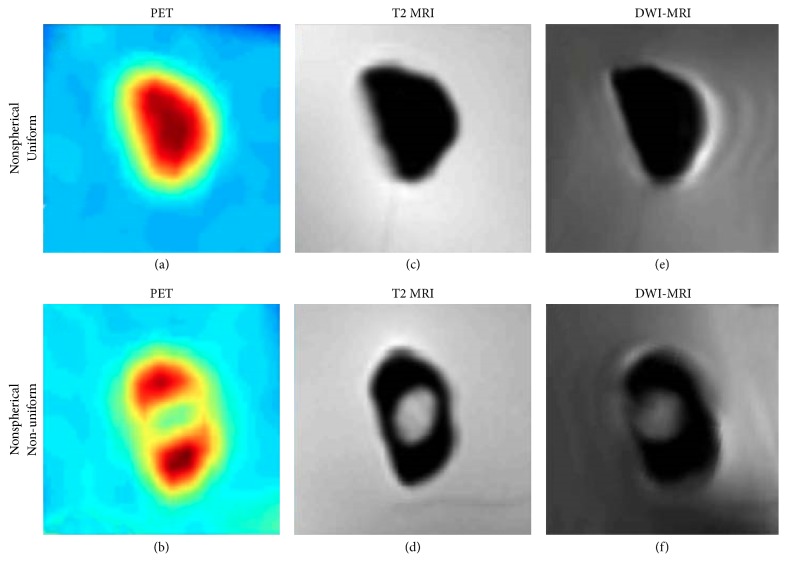
(a, c, e) PET and MR images of a nonspherical and uniform lesion; (b, d, f) PET and MR images of a lesion with simulated necrosis; (a-b) PET images; (c-d) T2-weighted image; (e-f) DW image with *b* = 0 s/mm^2^.

Figure 4 shows PET and MRI images obtained in a nonspherical lesion with uniform radioactive uptake (a, c, e) and in a nonspherical lesion with nonuniform radioactive uptake (necrosis is simulated) (b, d, f). PET, T2 MRI, and DW-MRI images are shown in Figures 4(a)-4(b), 4(c)-4(d), and 4(e)-4(f), respectively. From a qualitative evaluation, the lesions appeared as expected, and no artifacts, due to the Parafilm interface between the lesion and background, were visible.

From a quantitative point of view, considering lesion ADC, a percentage difference of −6%  ±  18% was estimated when ADC lesion volume was segmented by the fixed threshold segmentation method [13] and compared to ADC from calibration curves. Considering lesion radiotracer concentration, a percentage difference of 8%  ±  18% was found with respect to GS, demonstrating the feasibility of using agarose in PET for quantitation measurements.

These results highlight the phantom's feasibility and the potential for evaluating quantitative approaches in both PET and MRI-DW, also with necrotic portions within lesions.

### 3.3. Advantages and Limitations

In this work, a strategy to manufacture a dual-modality (DW-MRI and 18F-FDG PET) phantom, simulating realistic oncological conditions, was presented. The development of such phantoms was stimulated by the need to validate advanced MR and PET image processing approach which is a recognized need especially with the development of new/novel PET/MR imaging platforms. This developed strategy is feasible and easy, allowing manufacturing disposable and cheap phantoms simulating realistic conditions. Characterization of T2 relaxation properties showed that sucrose and agarose gels enable simulating T2 relaxation in breast tissues and calibration curves of ADC values as a function of sucrose concentration allowed to evaluate ranges of sucrose concentration to simulate malignant versus normal tissue. Furthermore, lesion manufacturing was compatible with the possibility of preparing a synthetic lesion with different gel mixtures simulating different tissue contributions. The procedure for phantom manufacturing was found to be feasible also for PET measurements even if for small amount of prepared gel, as in breast district simulation. The proof-of-concept study demonstrated how to use this phantom for validation of image processing strategies in PET and DWI-MR.

Despite this, there are some limitations that have to be considered in future developments. The manufactured phantoms are not really anthropomorphic, since the only realistic feature of the phantoms is the 3D modeling of the oncological lesions. Even if this could not impact MR acquisition in the quantitative validation of ADC values, the lack of an anthropomorphic phantom is particularly significant in PET where photon interactions and correction for gamma ray attenuation in human tissue surrounding the breast are not captured when using only two containers simulating breast without any background from thorax districts. Measurements in anthropomorphic phantoms, such as the anthropomorphic RSD Alderson Thorax phantom [14], with breast containers filled by a background gel mixture and realistic oncological lesions should be used to improve the accuracy of quantification in PET.

Furthermore, even if we demonstrated the feasibility of including different tissue types in the synthetic lesions, only a simplified heterogeneity inside tumor was achieved, simulating two tissue components, tumor and necrosis. The feasibility of manufacturing more complex situation should be addressed.

Moreover, the procedure we adopted could not allow accurately fixing the position of the necrotic core inside the tumor as well as the tumor in the scanner FOV. This may affect, for example, the replication/reproducibility of the measurements. Methodological improvements on the 3D-printing manufacturing procedure could be implemented by (1) 3D printing of 3D shells (tumors) including smaller and separate 3D shells with fixed shape, volume, and spatial position within the tumor, thus allowing more accurate positioning of necrotic cores inside the tumors, and (2) using a more rigid thin support to suspend the lesions within the phantom in order to better fix the lesion position and orientation into the phantom. Further measures could be also performed to specifically quantify stability over time of the tumor shape. Although we did not observe modifications in the shape of lesions over the acquisition time, we cannot exclude imperceptible shape modifications.

Another limitation is that our phantoms were developed mainly for research purposes, and in particular for validation of advanced image processing methods to be translated to clinical practice. Both in PET and in MR, there is the need of a realistic phantom to test the characteristics and accuracy of the scanner. The possibility of evaluating our cheap and disposable phantom for scanner's quality control should be evaluated since these kinds of phantoms are extremely valued and expensive too.

## 4. Conclusions

Our method is feasible for manufacturing anthropomorphic oncological phantoms and can be used for both ^18^F-FDG PET and DW-MRI quantification. The use of nonspherical containers and nonuniform materials and radioactivity concentrations makes these phantoms particularly realistic for simulating oncological lesions.

The method needs to be assessed in a wider range of clinical conditions, for example, in MRI with more compartments and with different diffusion properties for both cancer and normal tissues. Our phantom is inherently disposable and compatible with both PET and MRI acquisitions in a wide range of radioactivity and diffusion configurations. The characteristics shown in this work could also be particularly useful for manufacturing oncological phantoms compatible with both independent and simultaneous PET and MRI acquisitions using the new generation hybrid PET-MRI systems.
